# Uncovering Sequence and Structural Characteristics of Fungal Expansin‐Related Proteins With Potential to Drive Substrate Targeting

**DOI:** 10.1002/prot.70029

**Published:** 2025-08-01

**Authors:** Anna Pohto, Taru Koitto, Deepika Dahiya, Alessandra Castro, Elizaveta Sidorova, Martina Huusela, Scott E. Baker, Adrian Tsang, Emma Master

**Affiliations:** ^1^ Department of Bioproducts and Biosystems Aalto University Espoo Finland; ^2^ Department of Chemical Engineering and Applied Chemistry University of Toronto Toronto Canada; ^3^ Microbial Molecular Phenotyping Group Environmental Molecular Sciences Division, Earth and Biological Sciences Directorate, Pacific Northwest National Laboratory Washington USA; ^4^ DOE Joint Bioenergy Institute Emeryville California USA; ^5^ Centre for Structural and Functional Genomics, Concordia University Quebec Canada

**Keywords:** chitin, expansin, lignocellulose, loosenin, principal component analysis

## Abstract

Expansins loosen plant cell wall networks through disrupting non‐covalent bonds between cellulose microfibrils and matrix polysaccharides. Whereas expansins were first discovered in plants, expansin‐related proteins have since been identified in bacteria and fungi. The biological function of microbial expansins remains unclear; however, several studies have shown distinct binding preferences toward different structural polysaccharides. Earlier studies of bacterial expansin‐related proteins uncovered sequence and structural features that correlate to substrate binding. Herein, 20 fungal expansin‐related sequences were recombinantly produced in *Komagataella phaffii*, and the purified proteins were compared in terms of substrate binding to cellulosic and chitinous substrates. The impact of pH on the zeta potential of prioritized substrates was also measured, and Principal Component Analysis was performed to uncover correlations between protein characteristics (e.g., pI, hydrophobicity, surface charge distribution) and measured substrate binding preferences. Whereas acidic proteins with a predicted pI less than 5.0 preferentially bound to chitin, basic proteins with pI greater than 8.0 preferentially bound to xylan and xylan‐containing fiber. Similar to many cellulases, binding to cellulose was correlated to relatively high aromatic amino acid content in the protein sequence and presence of a carbohydrate binding module (CBM), which in the case of expansins is a C‐terminal CBM63. Whereas overall sequence characteristics could be correlated to substrate binding preference, the identity of amino acids occupying conserved positions that impact protein activity was better correlated with loosenin versus expansin classifications.

## 1 Introduction

Plant expansins contribute to acid‐induced plant cell wall growth, where they trigger cell wall loosening via a non‐lytic mode of action, possibly by disrupting non‐covalent interactions between matrix polysaccharides and cellulose microfibrils or at junctions between neighboring cellulose microfibrils [1, 2]. The broader biological significance of expansins has been underscored by genome sequencing, which uncovered expansins across land plants and expansin‐like proteins (EXLXs) within diverse microorganisms, including fungi and bacteria [3]. Both plant expansins and microbial EXLXs comprise a conserved two‐domain structure, consisting of an N‐terminal six‐stranded double‐Ѱ beta‐barrel (DPBB) domain (D1) resembling glycoside hydrolases from family 45 (GH45) but lacking a required catalytic residue, connected through a short linker to a C‐terminal domain (D2) that is classified as a family 63 carbohydrate‐binding module (CBM63) [4]. Besides plant expansins and microbial EXLXs, additional microbial expansin‐related proteins include swollenins such as SWO1 from *Trichoderma reesei* [5], which comprises a CBM1 and fibronectin domain at the N‐terminal end of the core EXLX two‐domain structure, and loosenins and ceratoplatanins that comprise only the D1 domain [6, 7].

Molecular modeling of plant expansins underscores the conservation of an extended groove connecting D1 and D2 domains, a “histidine‐phenylalanine‐aspartic acid” motif in D1 that defines the catalytic centre, and a glycine‐rich loop near the catalytic centre that can interact with bound substrates [8, 9, 10, 11]. Challenges relating to the recombinant production of plant expansins, however, have made it difficult to conduct detailed functional characterizations of this protein family. Instead, microbial expansin‐related proteins have drawn more recent attention, where applications have focused on promoting the enzymatic deconstruction of lignocelluloses to fermentable sugars [12, 13] and enabling the production of higher value cellulosic materials [14, 15]. In particular, the bacterial expansin‐like protein from *Bacillus subtilis* (*Bs*EXLX1) has been extensively studied. *Bs*EXLX1 has been shown to weaken cellulose filter paper, induce plant cell wall creep albeit to lesser extents than plant expansins, and preferentially bind hemicelluloses (e.g., xyloglucan, xylan) over cellulose [4, 16, 17]. The relative ease of recombinantly producing *Bs*EXLX1 in *Escherichia coli* has facilitated structural and site‐directed mutagenesis studies that have helped elucidate the molecular basis for substrate targeting and action of the protein [4, 16, 18]. Overall, the studies reveal the critical importance of Asp82 and the supporting function of Thr14, along with Ser16, Asp71, and Glu75, in mediating a *Bs*EXLX1‐driven glycan twist predicted to destabilize cellulosic structures [16, 18]. Corresponding studies also point to an indirect role for Thr12 and Tyr73 in stabilizing the Asp82‐Thr14 active centre of the *Bs*EXLX1 D1 domain. The combination of these conserved residues located along the groove spanning D1 and D2 forms a polysaccharide binding surface (PBS), which has been termed the front side of the protein [4, 19]. The potential relevance of protein surface charge distribution to substrate targeting motivated Pastor et al. [19] to compare the electrostatic polarization of front and back protein surfaces of 44 bacterial expansins. Notably, 40 of the 44 sequences were predicted as either acidic or basic proteins at pH 7.0, which suggests substrate targeting could be mediated through electrostatic interactions; substrate binding profiles, however, were not experimentally determined [19].

Only a handful of fungal expansin‐related protein structures have been experimentally determined, including *Tl*EXLX1 from *Talaromyces leycettanus* (7WVR) and *Pca*LOOL12 from *Phanerochaete carnosa* (9CE9) [13, 20]. To accelerate the elucidation of biological functions and applied potential of fungal expansin‐related proteins, sequence‐functional studies that include proteins with different modular structures are needed. Herein, we shed light on correlations between surface charge distribution, identity of conserved amino acid positions, modular structure, and experimentally determined binding profiles of 20 fungal proteins from diverse taxonomic origins. Since some microbial expansin‐related proteins preferentially bind to chitin over lignocellulosic substrates [21, 22, 23], pointing to possible interactions of these proteins with fungal cell walls [21, 22, 23], the binding studies performed herein included both plant polysaccharides as well as commercial alpha‐chitin. The correlation analyses then used Principal Component Analysis (PCA), which reduces multidimensional data into a two‐dimensional biplot and identifies parameters that introduce the most variability to the dataset. The parameters are represented as vectors such that proteins with the most similar parameter weightings cluster on the PCA plot. As was observed for bacterial sequences, the fungal sequences were divided into acidic versus basic proteins, and surface charge was correlated to binding to lignocellulosic versus chitinous substrates. The importance of the D2 domain to cellulose binding was also observed.

## 2 Materials and Methods

### 2.1 Materials

Avicel PH‐101 (50‐mm particle size; catalog no. 11365), xylan from oat spelt, and chitin from shrimp shells (CAS: 1398‐61‐4) were purchased from Sigma‐Aldrich. Oven‐dried (60°C) birch hardwood kraft pulp (98% dry matter content) was kindly provided by UPM‐Kymmene Oyj (Lappeenranta, Finland).

### 2.2 Sequence Selection and Recombinant Protein Production

The 20 fungal expansin‐related proteins used in this study were obtained from fungal genomes published in the Mycocosm database (https://mycocosm.jgi.doe.gov/mycocosm/home); selected sequences were synthesized and cloned into the pPICZalphaA vector by the Joint Genome Institute (Data S1). Except for the loosenins encoded by *Phanerochaete carnosa* (i.e., *Pca*LOOLs), which were recombinantly produced in *Komagataella phaffii* (previously *Pichia pastoris*) strain SMD1168H, all other expansin‐related proteins characterized herein were recombinantly produced in *Komagataella phaffii* strain KM71H in accordance with the manufacturer's instructions (Invitrogen, Thermo Fisher Scientific) and as previously described [12, 22]. Briefly, transformants were grown on YPD‐agar containing 100 μg/mL of Zeocin before being transferred to 500 mL buffered glycerol complex medium (BMGY) in 2 L non‐baffled Erlenmeyer flasks. The *K. phaffii* cultivations were grown at 30°C and 200 rpm until reaching an OD_600nm_ between 4 and 6. The cells were harvested by centrifugation and the cell pellets were suspended in 100 mL buffered methanol complex medium (BMMY) in 500‐mL Erlenmeyer flasks. The cultivations were induced with up to 3% methanol for 4 days at 20°C and at 160 rpm before recovering the supernatant by centrifugation and filtration through a 0.45 μm polyethersulfone (PES) filter (Millipore). The pH of the filtrate was adjusted to pH 7.8 using 2 M NaOH and filtered through a 0.45 μm PES filter before the recombinant 6xHis‐tagged proteins were purified by affinity chromatography using Ni‐NTA resin (Qiagen, #30230) [22]. Purified protein samples were exchanged to a storage buffer (Data S1) and concentrated using Vivaspin 20 Ultrafiltration Units (5 kDa and 10 kDa) from Sartorius. To increase the protein yield, some proteins were produced in a bioreactor according to Dahiya et al. [12] Details of each protein production are summarized in Data S2.

### 2.3 Analysis of Fungal Expansin‐Related Protein Sequences

Full protein sequences were used to generate identity matrices; N‐terminal signal peptides were predicted using SignalP versions 5 and 6 [24, 25] and trimmed from the sequence for all other sequence analyses. Protein sequence alignments used for the sequence identity matrix and sequence logos were then constructed using the EMBL‐EBI Clustal Omega MSA [26]. Identity matrices for D1 and D2 domains were constructed based on AlphaFold2 model predictions whereby the linker sequence was included with the D2 domain. Web logos were then created using the WebLogo 3 application [27, 28]. To examine structurally conserved key amino acids, the AlphaFold2 model of each protein was superimposed on the *Bs*EXLX1 solved structure (PDB 3D30). The matching structural position of each key residue in each AlphaFold2 protein model was checked and recorded.

The predicted pI and molecular weight values were calculated using computational tools available at Expasy.org. The values termed protein aromaticity and hydrophobicity refer to averages calculated from the protein sequences using the ProteinAnalysis class in the Bio.SeqUtils.ProtParam module of BioPython. Aromaticity was calculated based on the relative frequency of aromatic amino acids [29]. Hydrophobicity was calculated by assigning each amino acid a value from a pre‐established scale [30], after which an average of all the values in the sequence was calculated and used. For this measure, a higher value indicates a more hydrophobic protein. Protein charge values were calculated according to the method detailed by Pastor et al. [19] Briefly, amino acids with electrically charged side chains were calculated, whereby negatively charged amino acids at pH 5 (arginine, histidine and lysine) were each assigned a charge of –1e, and positively charged amino acids (aspartic acid and glutamic acid) a charge of +1e each. N‐ or C‐terminal extensions were not included in the charge calculations, as the goal was to compare only the D1 and D2 regions of the proteins.

### 2.4 Binding Studies

Protein binding to selected substrates was measured using the pull‐down assay described in Monschein et al. [22] Samples (500 μL) were prepared in 50 mM sodium acetate (pH 5.0) and comprised 12.5 mg substrate (2.5% w/v) and 100 μg/mL protein. Eppendorf LoBind microcentrifuge tubes (1.5 mL, Sigma‐Aldrich) were used for the protein binding experiments. Protein references were prepared using protein solutions without substrate; substrate references contained substrate suspensions without protein. For microcrystalline cellulose (Avicel), chitin, and xylan, the samples were incubated for 1 h at room temperature on a tube rotator set to 20 rpm. For hardwood pulp, the samples were incubated for 1.5 h using a thermoshaker (ThermoMixer C, Eppendorf) at 25°C and 1100 rpm. Supernatants were then recovered by centrifugation (15 000 rpm for 10 min) and the concentration of unbound protein was determined by BCA (Pierce BCA protein assay kit) using BSA (Sigma‐Aldrich; catalog no. A3059) as a reference. The zeta potential of substrates used in the binding studies was measured in 50 mM sodium acetate (pH 5.0) using a Malvern Zetasizer Nano ZS90.

### 2.5 Principal Component Analysis

The PCA and density‐based clustering were conducted with R. PCA was conducted with the prcomp function and visualized with the fviz_pca_biplot function. Protein characteristics included in the PCA were predicted pI value, protein charge, hydrophobicity, aromaticity, and substrate binding data. *Z*‐score normalization of the data were done prior to the PCA. Clustering of the PCA biplot data points were then performed using dbscan with an eps value (neighborhood radius of each cluster) of 0.75 and MinPts value (minimum number of data points in each cluster) of 1.

### 2.6 Protein Modeling

The construction of AlphaFold2 models was done using the ColabFold platform [31], which utilizes MMseqs2 search for faster single predictions. The best model constructed for each sequence was used. Models of predicted surface charge distribution were constructed with the AlphaFold2 models on the APBS‐PDB2PQR web server at pH 5.0 [32]. Comparison of models and modeling of disulfide bridges was conducted with the PyMOL software.

## 3 Results and Discussion

### 3.1 Comparative Analysis of Fungal Expansin‐Related Protein Sequences Selected for Recombinant Production

The 20 fungal expansin‐related proteins compared herein were recombinantly produced in *K. phaffii* to varying levels, from 2.7 to 393 mg/L (Data S2). The protein selection covered both structural and phylogenetic diversity. For example, seven of the proteins comprise only the D1 domain and thus are classified as loosenins. Whereas nearly half (nine) of the sequences originate from Basidiomycota, six originate from Ascomycota, and the remaining five originate from Mucoromycota, Zygomycota, Neocallimastigomycota, and Blastocladiomycota.

**FIGURE 1:**
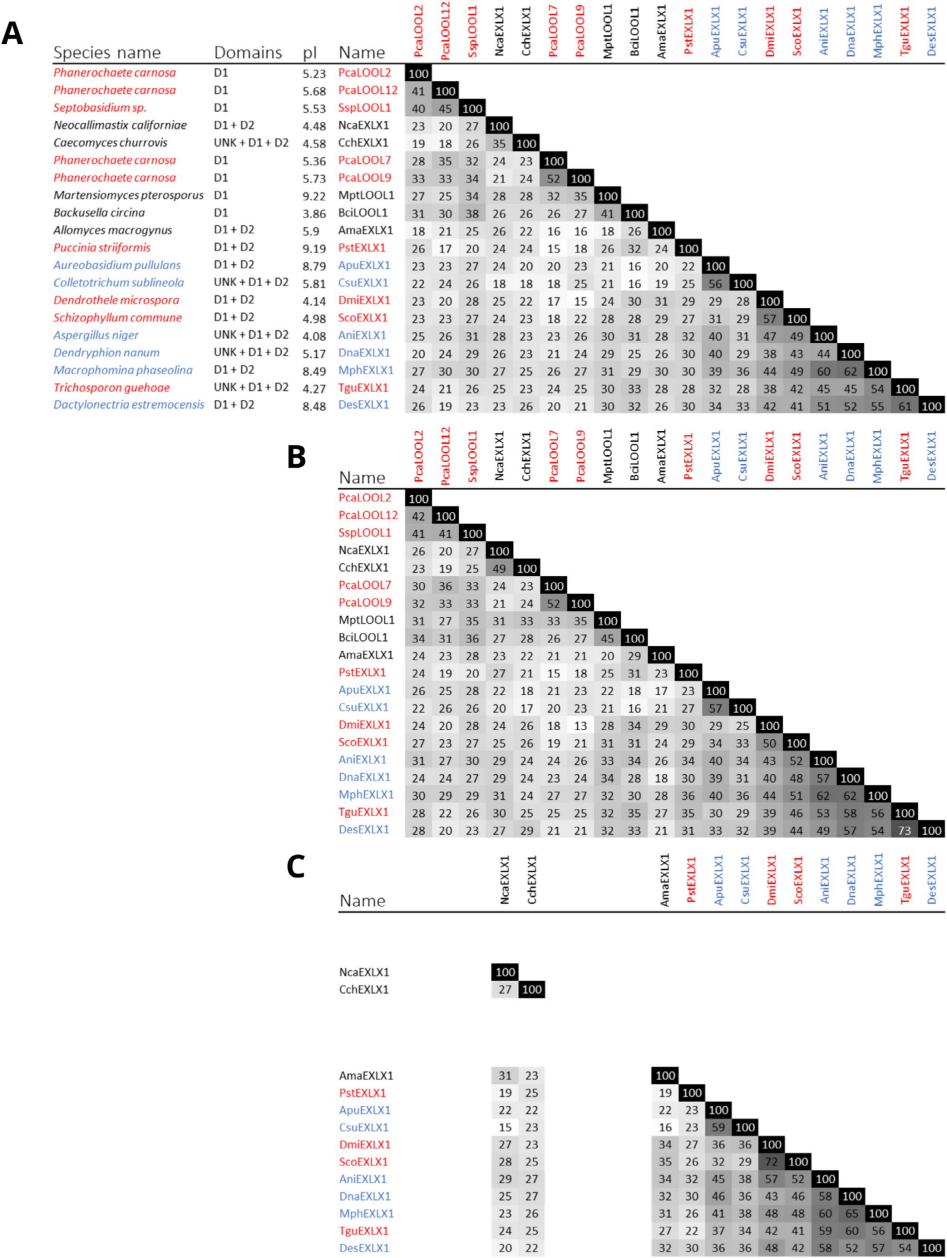
Identity matrices of selected fungal expansin‐related proteins. Species names highlighted in blue are part of the Ascomycota phylum and those in red are from Basidiomycota. Species names in black are from diverse fungal phyla (detailed in Data S1). (A) Percentage identity matrix of the whole sequences. (B) Percentage identity matrix of D1 of proteins. (C) Percentage identity matrix of D2 of proteins.

The predicted molecular weight of the selected proteins ranged from 10.4 to 50.2 kDa, and predicted pI values ranged from 3.75 to 9.4. Highest overall sequence identities were approximately 60% and shared among very few proteins from the Ascomycota phylum (Figure 1A); moreover, overall sequence identities among the EXLXs were spread across both D1 and D2 protein domains (Figure 1B,C). When considering the D1 and D2 domains separately, however, higher sequence identities (30%–52%) could be seen among loosenins as compared to between loosenins and the D1 domains of EXLXs (13%–35%) (Figure 1B). Among the EXLXs, most of the D1 and D2 domains were connected via a short linker (8–10 amino acids), with the exception of *Cch*EXLX1 and *Nca*EXLX1, whose linkers were double in length (19 amino acids).

Using PyMOL, five possible disulfide bridge positions were predicted among the fungal sequences, where each sequence included a total of 1–3 disulfide bridges (Data S1). Pastor et al. [19] define three sequence clades, where one of the defining factors is the number of disulfide bridges. Two disulfide bridges were shown to distinguish the bacterial expansins in clades 1 and 2 from those in clade 3. Clade 3 sequences lack disulfide bridges altogether and include *Bs*EXLX1 [19]. Two of the disulfide bridges conserved in the clade 1 and 2 bacterial sequences that are present in the fungal expansins studied herein are referred to as disulfide bridge A and B. Disulfide bridge A is highly conserved among green plants and fungi [33] and is present in all of the fungal sequences studied herein. Disulfide bridge B is positioned in a loop close to the catalytic aspartic acid (i.e., Asp82 by *Bs*EXLX1 numbering). It is predicted in plant EXPA but not EXPB expansins [11] and is present in all the fungal sequences studied herein except *Apu*EXLX1, *Pst*EXLX1, *Csu*EXLX1, and *Ama*EXLX1. Notably, disulfide bridge B is also present in the GH45 subfamily C enzymes. Relative to other GH45 family members, those from subfamily C can adopt an extended substrate binding site [34]; similarly, the presence of the loop containing disulfide bridge B is predicted to affect the PBS in expansins [11]. Over half of the fungal proteins were predicted to have three disulfide bridges; most of these were EXLX sequences comprising both D1 and D2 domains; however, loosenin *Mpt*LOOL1 is also predicted to comprise three disulfide bridges. In these cases, two of the three disulfide bridges were always disulfide bridge A and C. Disulfide bridge C is conserved in GH45 enzymes, connects the core of D1 to the beginning of the linker sequence, and was only predicted in fungal sequences expected to form three disulfide bridges [11, 33] Disulfide bridge D and E were comparatively rare, where disulfide bridge D is located near the N‐terminus of the protein and disulfide bridge E is the only disulfide bridge predicted in the D2 domain.

### 3.2 Fungal Expansin‐Related Proteins Reveal Different Substrate Binding Preferences

**FIGURE 2:**
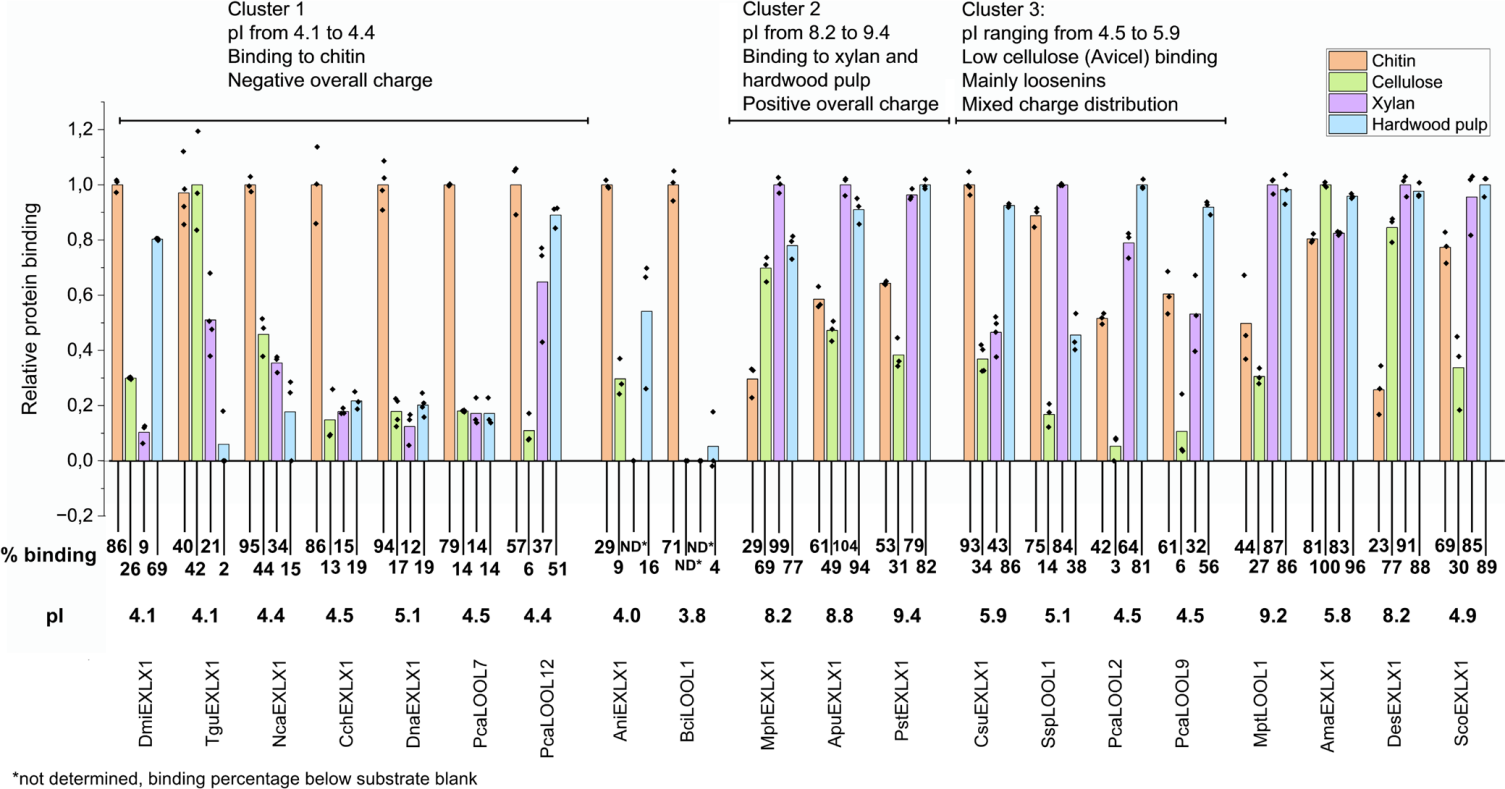
Binding by recombinant fungal expansin‐related proteins to selected substrates at pH 5.0; chitin (orange), microcrystalline cellulose (Avicel, green), oatspelt xylan (purple), hardwood kraft pulp (blue). The substrate binding results were normalized where the substrate leading to highest protein binding was assigned a value of 1.0. The corresponding average percent binding values are reported below each protein, as well as in Data S3. Percentage binding that was below the substrate blank value was not determined, marked ND. Protein predicted pI values are also listed. Proteins are grouped based on similar binding patterns. Binding experiments were performed at least in triplicate, and individual binding values for each protein are as black diamonds above each substrate.

Earlier studies report microbial expansin‐related proteins with different binding preferences towards cellulose and plant matrix polysaccharides such as hemicelluloses and pectin. For example, Olarte‐Lozano et al. [35] compare the basic *Bs*EXLX1 and the acidic *Pc*Exl1 from *Pectobacterium carotovorum* , showing preferential binding of *Bs*EXLX1 and *Pc*Exl1 to pectin and cellulose components of plant cell walls, respectively. More recent studies that measured the binding of microbial expansin‐related proteins to cellulosic versus chitinous substrates report preferential binding by acidic proteins to chitin substrates [21, 22]. To further investigate correlations between protein pI and substrate binding, the 20 recombinant fungal expansin‐related proteins produced herein were tested for binding to microcrystalline cellulose (Avicel), oat spelt xylan, hardwood pulp, and chitin (Figure 2; Data S3).

**FIGURE 3:**
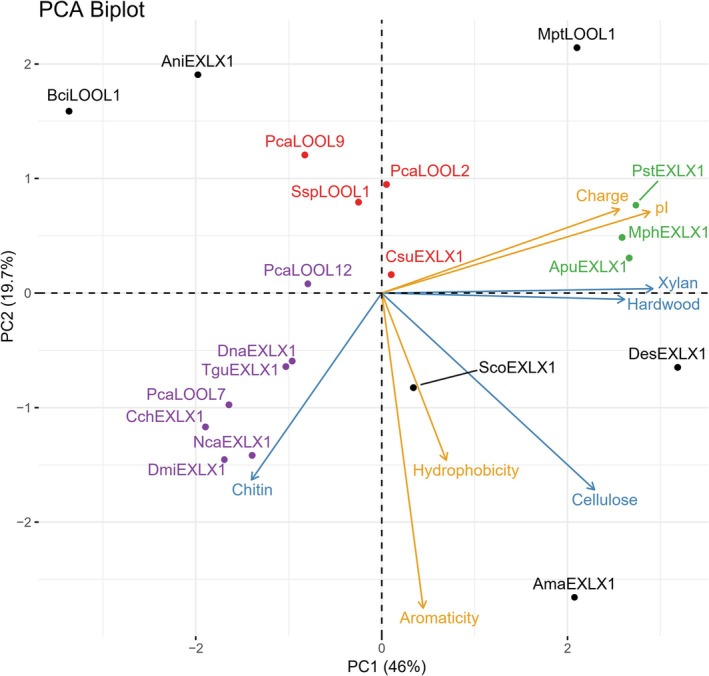
Principal component analysis of protein parameters. The loading scores for principal component 1 (PC1) and principal component 2 (PC2) for sequence‐based features are highlighted in yellow and experimentally determined binding percentages to different substrates in blue. Density‐based clusters with more than one member are highlighted in different colors: Cluster 1 in purple, Cluster 2 in green and Cluster 3 in red.

PCA was then used to uncover trends among expansin‐related sequences and substrate binding preference. In addition to the substrate binding profiles, the PCA considered multiple sequence features including predicted pI, overall charge, aromaticity, and hydrophobicity (Data S4). A principal component (PC) determines the linear combinations for each protein sample in regard to the different measured or calculated protein parameters. The amount of variability in the data are described by a percentage for each PC, the total of all PCs equaling 100%. In this case, PC1 and PC2 account for most of the variability, at 46% and 19.7%, respectively. Therefore, by looking at the PC1 and PC2 biplot (Figure 3) alone, sample clusters can be determined based on the parameters that affect the samples the most. The loading scores in the biplot show how each parameter contributes to the variation in the data and are represented by vectors that drive the individual sample placement on the PCA biplot (Figure 3; Data S4). Therefore, the samples that cluster together have similar values in those parameters that introduce the most variability in the data overall.

Several trends emerged from the PCA analysis that inform sequence characteristics likely to impact substrate binding. First, the PCA biplot clearly differentiated proteins that preferentially bound to chitin versus lignocellulosic substrates by protein pI, where having a comparatively low pI was correlated to comparatively high binding to chitin (Figure 3). By contrast, proteins that bound well to cellulose tended to have high aromaticity and hydrophobicity values (Figure 3). Briefly, aromaticity is calculated based on the frequency of tryptophan, phenylalanine, and tyrosine in the amino acid sequence [29], whereas hydrophobicity refers to an overall score calculated from Roseman scale hydrophobicity values [30]. Notably, loosenins were distributed across the biplot except for the quadrant that grouped expansin‐related proteins that bound well to cellulose. This observation underscores the particular importance of the D2 domain to cellulose binding [16].

Density‐based clustering of points on the PCA was then performed to identify proteins that act most similarly to one another. Three clusters with more than one member were formed: Cluster 1 contained *Dna*EXLX1, *Tgu*EXLX1, *Cch*EXLX1, *Nca*EXLX1, *Dmi*EXLX1, *Pca*LOOL7, and *Pca*LOOL12; Cluster 2 contained *Pst*EXLX1, *Mph*EXLX1, and *Apu*EXLX1; and Cluster 3 contained *Pca*LOOL9, *Pca*LOOL2, *Ssp*LOOL1, and *Csu*EXLX1. These clusters group in different regions of the PCA biplot, suggesting similar behavior of proteins within a cluster and different behavior of proteins within different clusters. Proteins in Cluster 1 showed preferential binding to chitin over other tested substrates (Figure 2). The pI values of proteins in Cluster 1 were approximately 5.0 or less; thus, these proteins are expected to be negatively charged when used in the binding assay. At the same time, the zeta potential value of the chitin substrate is positive at pH 5.0 (Data S5). Together, these results suggest electrostatic interactions are especially important for protein binding to chitin. Proteins in Cluster 2 displayed high binding to xylan and hardwood pulp. The pI of proteins in Cluster 2 is above pH 5.0, and so these proteins are expected to be positively charged when used in the binding assay. Since the zeta potential value of xylan and hardwood pulp is negative at pH 5.0, protein binding to these substrates is also likely driven by electrostatic interactions, albeit opposite to those mediating chitin binding (Data S5). Whereas proteins in Clusters 1 and 2 are differentiated by calculated pI, proteins in Cluster 3 are mainly loosenins and are differentiated by having low aromaticity and low binding to cellulose. The clustering constraints applied herein led to 6 of the 20 fungal proteins not being grouped within one of the three protein clusters. Nevertheless, the binding preferences of these proteins were similarly correlated to the predicted pI and presence of D2, along with surface charge distribution, as described in the next section.

### 3.3 Correlation Between Protein Sequence and Predicted Structure With Substrate Binding

**FIGURE 4:**
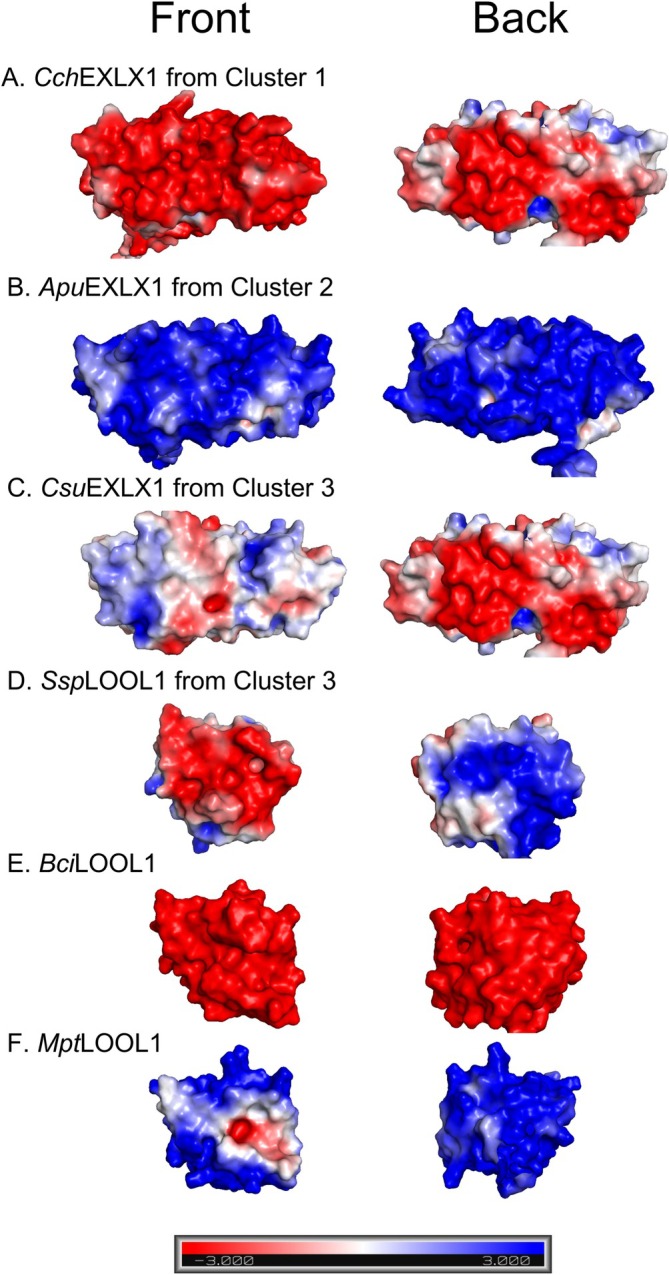
Predicted surface charge distribution of selected fungal expansin‐related sequences at pH 5.0. On the left is the “front” surface of the protein, which displays the polysaccharide binding surface (PBS). On the right is the “back” surface of the protein. The color scale is defined by a bar at the bottom of the figure and is from −3 to 3 keV. (A) *Cch*EXLX1 from Cluster 1. (B) *Apu*EXLX1 from Cluster 2. (C) *Csu*EXLX1 from Cluster 3. (D) *Ssp*LOOL1 from Cluster 3. (E) *Bci*LOOL1. (F) *Mpt*LOOL1. For some of the models, N‐ or C‐terminals have been further cropped for better visibility of the PBS.

Besides the presence of D2 correlating with binding to cellulose and low pI correlating with binding to chitin, differences in the distribution of charged amino acids at the protein surface are expected to impact substrate binding preference. In particular, it has been hypothesized that polarity between the PBS and the back surface of the protein can be important for substrate targeting [19, 35]. To investigate correlations between surface charge distribution and substrate binding by fungal expansin‐related proteins, model structures were constructed for the fungal proteins; pH 5.0 was assumed and then surface charge distribution in addition to the presence of conserved amino acids was investigated (Figure 4; Data S6).

Similar to bacterial EXLXs, nearly all of the fungal expansin‐related protein models showed a negative surface at the center of D1, which is formed by the aspartic acid, glutamic acid, and histidine residues that form the PBS [19]. As observed for acidic bacterial EXLXs, most of the acidic fungal proteins in Cluster 1 exhibited a comparatively negative front surface and positive or neutral back surface; moreover, like basic bacterial EXLXs, the basic fungal proteins in Cluster 2 as well as the basic *Des*EXLX1 exhibited a comparatively positive or neutral front surface and positive back surface (Figure 4; Data S6). Although the fungal proteins in Cluster 3 are acidic, they were grouped based on sharing low aromaticity and low binding to cellulose, which might explain why *Csu*EXLX1 and *Ssp*LOOL1 are both grouped in Cluster 3 and yet display reverse surface charge distributions (Data S6). *Ani*EXLX1, *Bci*LOOL1, and *Mpt*LOOL1 did not group into any of the three protein clusters and were differentiated by displaying a uniform surface charge. Whereas *Ani*EXLX1 and *Bci*LOOL1 are evenly negatively charged and showed especially low binding to xylan, *Mpt*LOOL1 is evenly positively charged and bound well to xylan (Data S3).

**FIGURE 5:**
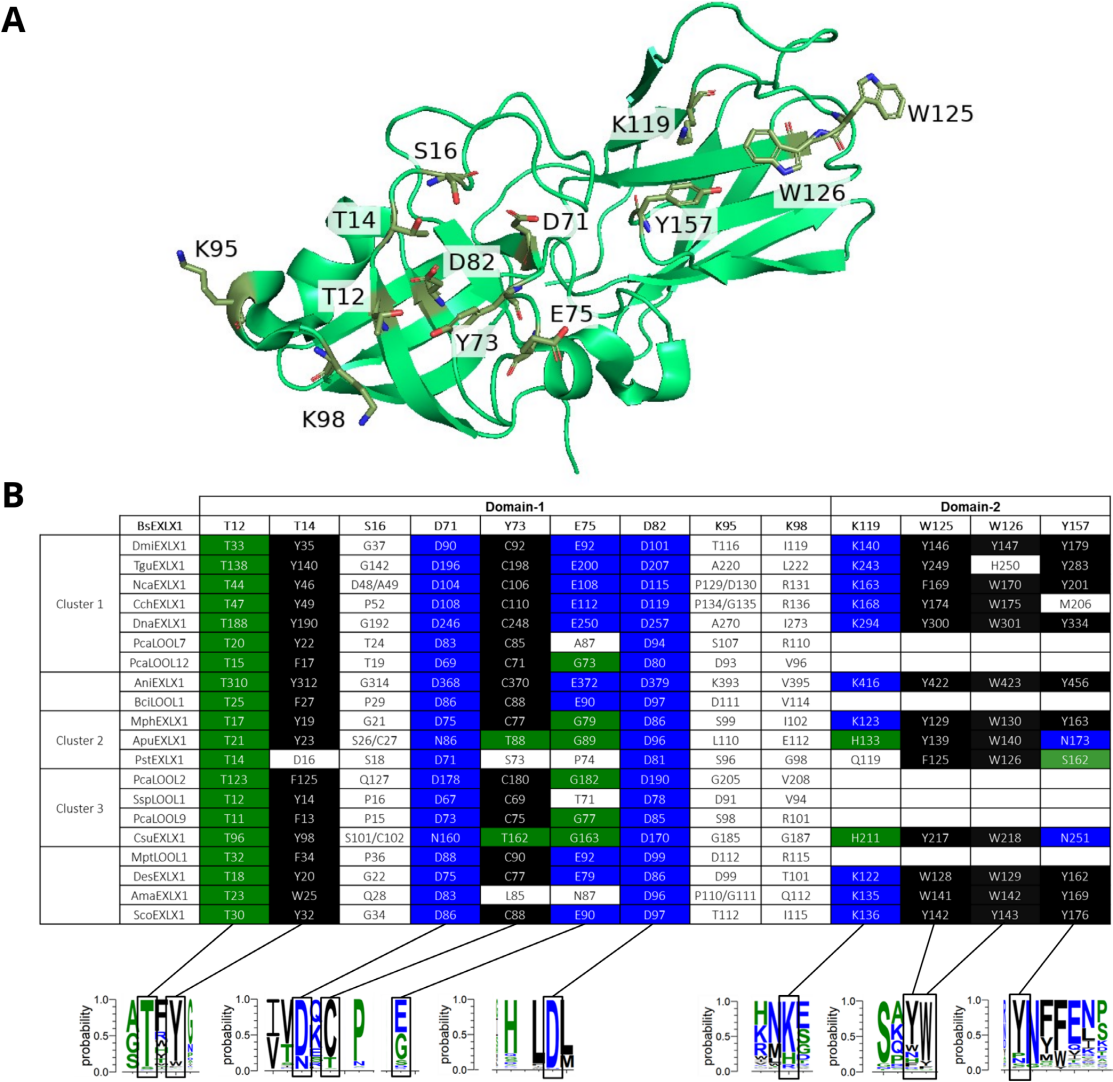
(A) Ribbon model of *Bs*EXLX1. *Bs*EXLX1 is constructed from a solved structure (PDB 3D30). Stick models of residues previously determined to be significant for *Bs*EXLX1 binding and activity are shown in the model. (B) Table comparing conservation of important *Bs*EXLX1 residues in the fungal sequences at the same positions. Below, a sequence logo of fungal residues is displayed, highlighting and connecting corresponding amino acids in the table in matching color.

Previous mutagenesis and molecular dynamics studies of the *Bs*EXLX1 investigated the role of amino acids largely conserved by bacterial expansin‐related proteins [16, 36]. A previous study by Ding et al. (2022) solved the first crystal structure of a fungal expansin *Tl*EXLX1 (PDB 7WVR) and explored crucial amino acids for its binding activity and synergism with a commercial cellulase, Cellic Ctec2. These findings were concurrent with the effect the corresponding amino acids have in *Bs*EXLX1. The occurrence of corresponding amino acids in the fungal homologs studied herein was evaluated and correlated to substrate binding; a sequence logo comprising over 1200 fungal expansin‐related proteins was also generated to identify amino acid substitutions that are conserved among the larger set of fungal sequences (Figure 5).

Asp82 in *Bs*EXLX1 is essential for protein function [16] and is conserved among all the fungal sequences. Mutagenesis and dynamic modeling studies suggest Asp82 in *Bs*EXLX1 plays a critical role in twisting and thereby destabilizing glycan structures [16, 18]. These same studies predict supporting roles for Thr14, Ser16, Asp71, and Glu75 in substrate twisting and in stabilizing Asp82 interactions with Thr12 and Tyr73 [18]. Among these amino acids that play a supporting role in *Bs*EXLX1 activity, only Thr12 is conserved among all the fungal sequences; Asp71 is also highly conserved with infrequent substitutions being largely to asparagine (Figure 5B). Substituting Thr14 or Tyr73 to alanine reduces *Bs*EXLX1 activity by 60% and over 90%, respectively [16]; however, previous studies comparing over 40 bacterial sequences indicate the roles of Thr14 and Tyr73 in *Bs*EXLX1 can be achieved through alternative sequence combinations. In the Pastor et al. [19] defined sequence clades, clade 3 has a tyrosine and threonine residue framing Asp82 in the D1 structure [18, 19]. In *Bs*EXLX1, these residues are Tyr73 and Thr14, respectively. The bacterial sequences in clades 1 and 2 appear to replace corresponding functions by switching a tyrosine to the position of the clade 3 threonine, and disulfide bridge B in place of the clade 3 tyrosine [19, 33]. Such substitutions were also observed in plant expansins [11, 33] and nearly all the fungal EXLXs studied herein. Of note, five of the six loosenins included in this study replaced Thr14 with phenylalanine rather than tyrosine, which invites the question of whether in the absence of a D2 domain, substrate interactions in loosenins could be further stabilized through interactions with the less polar phenylalanine residue in D1. In contrast to the considerable impact of positions Thr14 and Tyr73 to *Bs*EXLX1 activity, mutagenesis of Ser16 and Glu75 led to moderate (30%–39%) reductions in the activity of the protein [16]. Consistent with this finding, the fungal expansin‐related sequences appeared to lack conservation at the position equivalent to Ser16. On the other hand, the most notable difference in functional amino acids that correlated with PCA cluster was the absence in Clusters 2 and 3 of a glutamic acid at the position equivalent to Glu75 in *Bs*EXLX1.

Site‐directed mutagenesis of the D2 domain of *Bs*EXLX1 confirmed the importance of three aromatic amino acids Trp125, Trp126, and Tyr157 as well as Lys119 to forming the polysaccharide‐binding surface along the front side of *Bs*EXLX1, which channels the polysaccharide substrate to the Asp82 catalytic residue [16, 37]. Lys119 is thought to further stabilize substrate binding by forming a hydrogen bond with the glycan chain. These same amino acids occupy corresponding positions in over 70% of the fungal sequences studied herein. Whereas substitutions of Trp125, Trp126, and Tyr157 were to another aromatic amino acid, the few substitutions of Lys119 were to histidine and glutamine. At pH 5.0, histidine is also positively charged and could form a hydrogen bond with the glycan chain. Still, substituting Lys119 with histidine or glutamine might not substantially affect cellulose binding as several fungal EXLXs that retained Lys119 showed comparatively low binding to cellulose. *Ama*EXLX1 and *Des*EXLX1 were the only recombinantly produced proteins with a tryptophan at both the Trp125 and Trp126 positions, which might explain their highest binding values (100% and 76%, respectively) to cellulose among all the proteins studied herein.

## 4 Conclusions

This comparative analysis of 20 diverse fungal expansin‐related proteins highlights the importance of electrostatic forces to substrate binding preference, where acidic proteins bound preferentially to chitin, and basic proteins bound preferentially to xylan and hardwood pulp. Although not sufficient for preferential binding to cellulose, high cellulose binding was shown to depend on the presence of the D2 domain. Whereas *Bs*EXLX1 from *Bacillus subtilis* is the most extensively characterized expansin to date, it is different from most other bacterial expansin‐like proteins (i.e., belonging to clades 1 and 2), plant expansins, and fungal expansin‐related proteins, which similarly comprise at least two disulfide bridges and often substitute Thr14 and Tyr73 in *Bs*EXLX1 for a tyrosine and disulfide bond, respectively. Such differences relative to *Bs*EXLX1, in addition to new substrate binding data and solved structures for other microbial expansin‐related proteins (e.g., PDB 9CE9), motivate additional molecular dynamics simulations to deepen our understanding of the molecular basis for expansin activity. In the meantime, the impressive variety of protein surface charge distributions presented by the diverse fungal expansin‐related proteins studied herein can guide comparative functional studies that hasten our discovery of distinct biological roles and applications for different microbial expansin‐related proteins.

## Author Contributions

**Anna Pohto:** conceptualization, formal analysis, investigation, software, visualization, writing – original draft, writing – review and editing. **Taru Koitto:** formal analysis, investigation, writing – review and editing. **Deepika Dahiya:** formal analysis, investigation, writing – review and editing. **Alessandra Castro:** formal analysis, investigation, writing – review and editing. **Elizaveta Sidorova:** formal analysis, investigation, writing – review and editing. **Martina Huusela:** formal analysis, investigation, writing – review and editing. **Scott E. Baker:** data curation, resources, writing – review and editing. **Adrian Tsang:** data curation, project administration, resources, supervision, writing – review and editing. **Emma Master:** conceptualization, data curation, funding acquisition, project administration, supervision, writing – original draft, writing – review and editing.

## Conflicts of Interest

The authors declare no conflicts of interest.

## Supporting information


**Data S1:** Details of the selected protein sequences.

**Data S2:** Production and yield of recombinantly produced proteins used in the study.

**Data S3:** Protein binding to selected substrates (raw data and calculations).

**Data S4:** Computational and Experimental Data for PCA.

**Data S5:** Zeta potential values of substrates used for protein binding.

**Data S6:** Surface charge distributions of the proteins used in the study.

## Data Availability

The data that supports the findings of this study are available in the Supporting Information of this article.
